# Supplementary material for: Endothelial gene regulatory elements associated with cardiopharyngeal lineage differentiation
Source: Commun Biol. 2024 Mar 21;7:351. doi: 10.1038/s42003-024-06017-8 (PMC10957928; doi:10.1038/s42003-024-06017-8)

Supplementary Figure 1

Uncropped pictures of gels shown in Fig. 1b

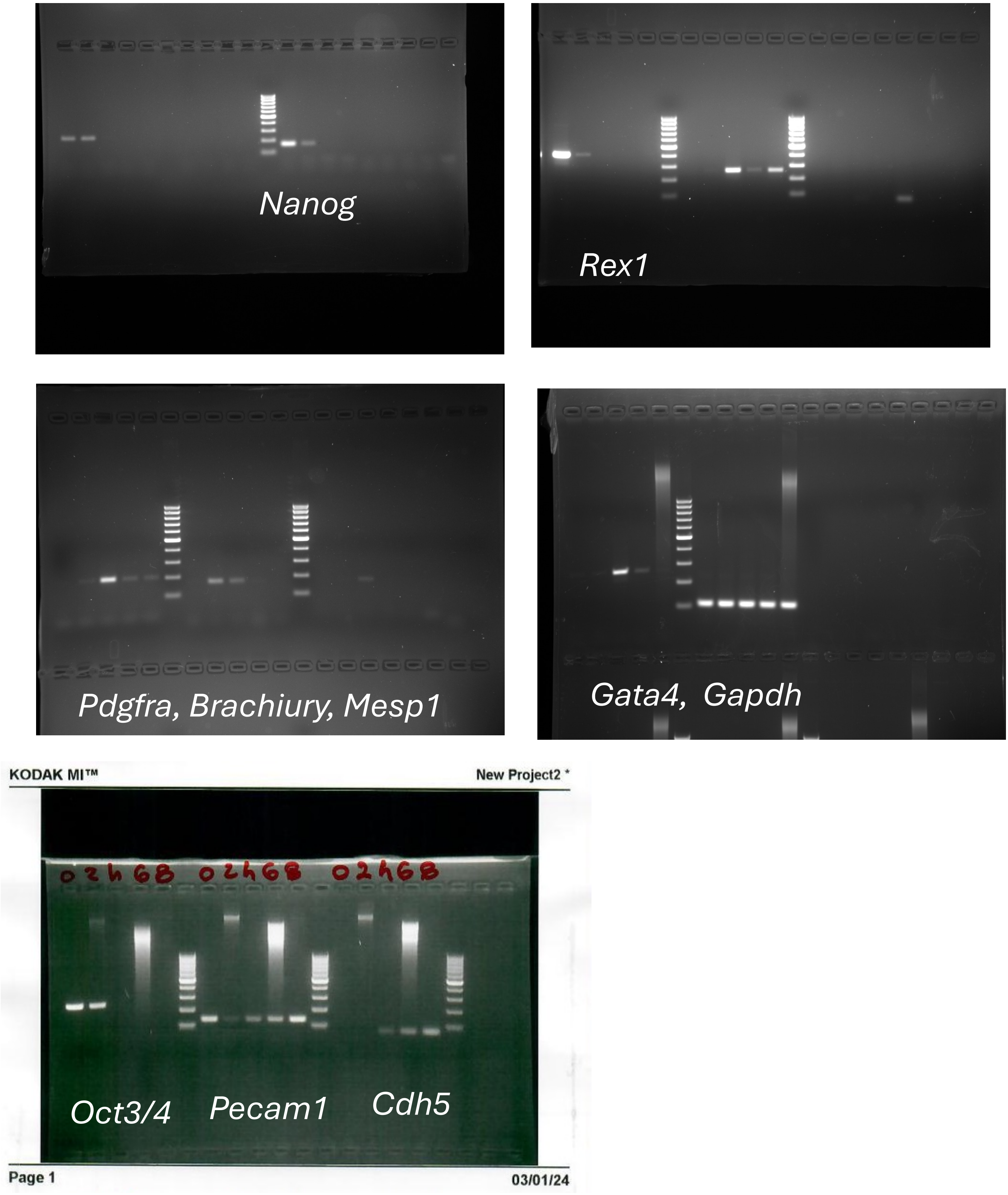

Supplementary Figure 1 (continued)

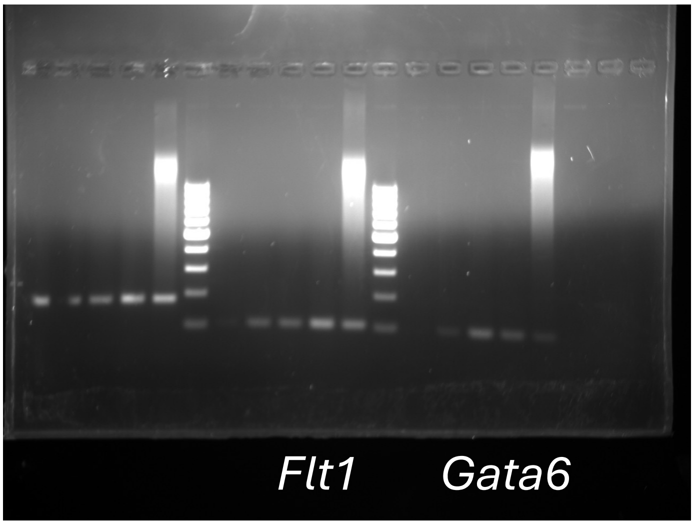

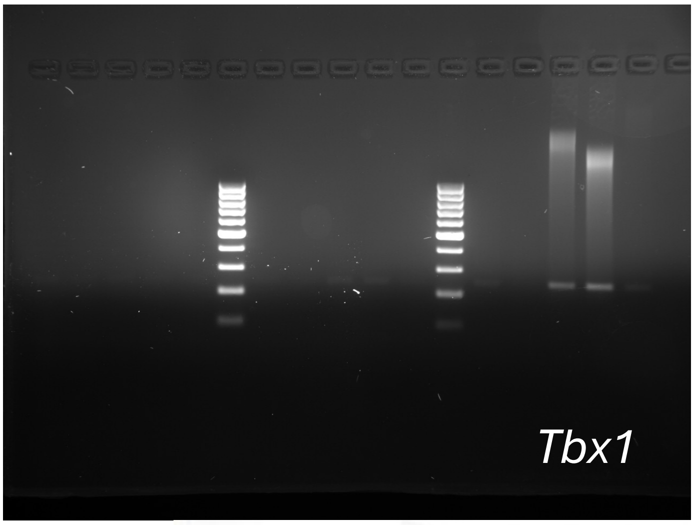

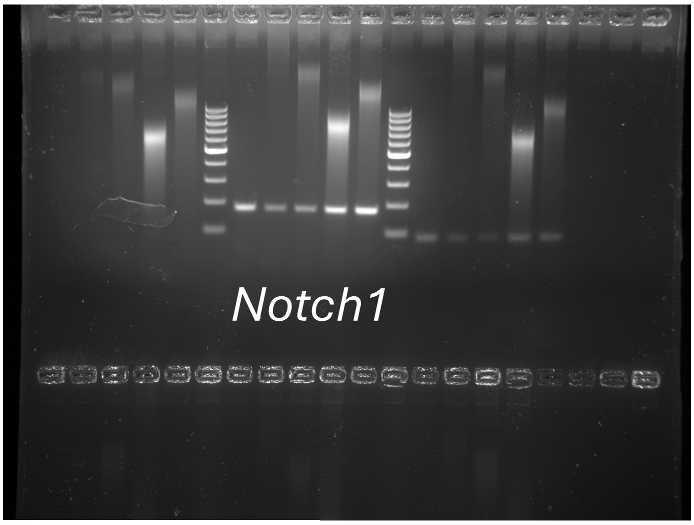

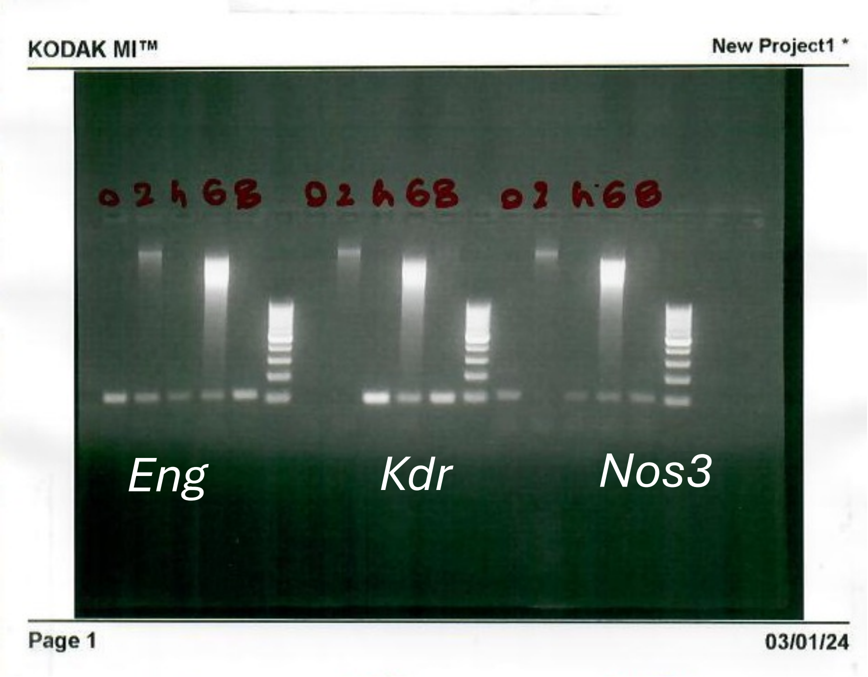

Supplement: Supplementary file 2 — Supplementary Information [file 42003_2024_6017_MOESM2_ESM.pdf]
