# Supplementary material for: Endothelial gene regulatory elements associated with cardiopharyngeal lineage differentiation
Source: Commun Biol. 2024 Mar 21;7:351. doi: 10.1038/s42003-024-06017-8 (PMC10957928; doi:10.1038/s42003-024-06017-8)
Supplement: Supplementary file 3 — Description of Additional Supplementary Files [file 42003_2024_6017_MOESM3_ESM.pdf]

Description of Additional Supplementary Files

File name: Supplementary Data 1
Description:
Sheet 1: Differentially expressed genes (d4 Vs d2). Positive values are for genes up regulated at d4.
Sheet 2: All expressed genes at d2 and at d4.
Sheet 3: GO_RNAseq_DEGs.
Sheet 4: GO_RNAseq_upregulated at d4.

File name: Supplementary Data 2
Description:
Sheet 1: Differentially accessible regions (DARs), d4 Vs d2. Positive values are for regions more accessible at d4.
Sheet 2: All consensus ATAC peaks at d2.
Sheet 3: All consensus ATAC peaks at d4.
Sheet 4: Common peak set (peakome).

File name: Supplementary Data 3
Description:
Sheet 1: 252 marker genes from Tbx1Cre EC cluster 6.
Sheet 2: 1217 marker genes from Mesp1Cre cluster c2.
Sheet 3: 1434 marker genes from Mesp1Cre cluster c16.
Sheet 4: 536 unique DARs mapped to EC marker genes from Mesp1Cre clusters.

File name: Supplementary Data 4
Description:
Sheet 1: Motifs *Tbx1*^{Cre} EC cluster 6.
Sheet 2: Motifs *Mesp1*^{Cre} EC cluster c2.
Sheet 3: Motifs *Mesp1*^{Cre} EC cluster c16.

File name: Supplementary Data 5
Description: Primers and gRNA sequences.

File name: Supplementary Data 6
Description: Source data for graphs and charts.
